# Regulation of PI3K/Akt dependent apoptotic markers during b virus infection of human and macaque fibroblasts

**DOI:** 10.1371/journal.pone.0178314

**Published:** 2017-05-30

**Authors:** Mugdha Vasireddi, Julia K. Hilliard

**Affiliations:** Viral Immunology Center, Biology Department, Georgia State University, Atlanta, GA, United States of America; Cornell University, UNITED STATES

## Abstract

B virus (*Macacine herpesvirus* 1), a simplex virus endemic in macaques, causes encephalitis, encephalomyelitis, and death in 80% of untreated zoonotically infected humans with delayed or no treatment. Here we report a significant difference in PI3K/Akt-dependent apoptosis between B virus infected human and macaque dermal fibroblasts. Our data show that B virus infection in either human or macaque fibroblasts results in activation of Akt via PI3K and this activation does not require viral *de novo* protein synthesis. Inhibition of PI3K with LY294002 results in a significant reduction of viral titers in B virus infected macaque and human fibroblasts with only a modest difference in the reduction of virus titers between the two cell types. We, therefore, tested the hypothesis that B virus results in the phosphorylation of Akt (S473), which prevents apoptosis, enhancing virus replication in B virus infected macaque dermal fibroblasts. We observed markers of intrinsic apoptosis when PI3K activation of Akt was inhibited in B virus infected macaque cells, while, these apoptotic markers were absent in B virus infected human fibroblasts under the same conditions. From these data we suggest that PI3K activates Akt in B virus infected macaque and human fibroblasts, but this enhances virus replication in macaque fibroblast cells by blocking apoptosis.

## Introduction

B virus (herpes B virus, *Macacine herpesvirus* 1), belongs to the family *Herpesviridae*, and is relatively innocuous in macaques where coevolution of the virus and host have transpired over millions of years [1, 2]. Zoonotic infection with B virus, on the other hand, results more commonly in severe disease with encephalitis, encephalomyelitis, ascending paralysis, and death in 80% of individuals without timely antiviral intervention [3, 4]. Co-evolution of a host and pathogen generally ensures selection of a kind of symbiosis between host and pathogen, whereas emergence of a pathogen in permissive foreign hosts can cause havoc in the absence of coevolution and selection opportunities. Understanding the differences in virus: host cell interactions between natural versus foreign hosts provides insight into mechanisms that can be exploited for development of effective therapeutics/interventions.

Virus infection of targeted cells triggers receptor-specific signaling pathways that have been selected throughout evolution to protect the host from foreign invaders, Each specific virus, however, has selected traits by which they counter specific cell defenses to ensure replication, transmission, and longevity. Phosphorylation of serine/threonine kinase Akt (protein kinase B/PKB) is an important activation event triggered by most viruses in order to block activated programmed cell death to facilitate virus infection. Upon stimulation of receptors, e.g., receptor tyrosine kinases (RTK) [5–9], integrins [10], and G-protein coupled receptors (GPCR) [11] etc., recruitment of phosphoinositol 3-kinase (PI3K) ensues [12, 13]. PI3K consists of two subunits, p85 and p110. Recruitment of PI3K occurs via SH2 binding of p85 subunit to phosphotyrosine residues on the receptors [14]. Activated PI3K phosphorylates membrane phospholipids, phosphatidylinositol 3,4-biphosphate (PIP2) to phosphatidylinositol 3,4,5- triphosphate (PIP3 [15]. Subsequently, PIP3 rapidly recruits cytosolic Akt and phosphoinositide-dependent kinase 1 (PDK 1) to the plasma membrane, where PDK1 phosphorylates Akt at threonine 308 (T308) [13, 16, 17]. This, plus the subsequent phosphorylation of serine 473 residue (S473) by PDK2 or other kinases, result in the activation of Akt. There are alternative pathways, as well, that activate Akt-independently of PI3K, including Ack 1, Src, PTK6, IKBKE, TBK, and others [18]. Activated Akt plays an important role in promoting cell survival, inhibiting apoptosis, protein synthesis, glycogen synthesis, and cell growth.

Many DNA and RNA viruses activate PI3K/Akt to ensure successful viral replication by forcing the cell to remain intact, including herpes viruses, which are known to activate PI3K/Akt signaling pathway for successful virus replication. Herpes simplex virus type 1 (HSV-1) activates Akt to block apoptosis in oral epithelial cells [19]. In case of HSV-1, not one but multiple viral proteins are involved in activating Akt in HSV-1 infected cells [20, 21] indicating Akt activation is an important event for HSV-1 replication and entry [22, 23]. Varicella zoster virus (VZV) infection also has been reported to induce PI3K-dependent activation of Akt via different ORFs, including ORF 12, ORF 63, and ORF 44 [24]. Beta herpes viruses, such human cytomegalovirus (HCMV) [25] and gamma herpesviruses, such as Epstein-Barr virus (EBV) [26, 27] and Kaposi-sarcoma associated herpesvirus [28], also activate Akt to maximize efficient virus infection.

Due to the significant role(s) of Akt activation during human herpesvirus infections, we hypothesized that B virus activates Akt in infected cells to drive efficient virus replication. Data from experiments presented here showed the induction of Akt phosphorylation in both human and macaque fibroblast cells. To further evaluate the role of Akt activation, we blocked activation to determine the effects of Akt phosphorylation in blocking apoptosis and increasing the efficiency of virus replication. From these data, we hypothesize that human fibroblast cells either do not sense the virus presence and/or the virus utilizes an alternative mechanism to force the cell to remain alive to support replication.

## Materials and methods

### Cells and virus

Human foreskin fibroblast cells (HFF, ATCC # CRL-2097) obtained from ATCC® (Manassas, VA) were maintained as monolayers in minimal essential media (MEM) purchased from Mediatech, Inc. (Manassas, VA) supplemented with 10% fetal bovine serum (FBS) (Atlanta Biologicals, Norcross, GA), 1% non-essential amino acids, and 1% sodium pyruvate. Rhesus macaque fibroblasts (RMF) were isolated (Brock et al., unpublished data) from dermal explants (provided through the Yerkes National Primate Research Center tissue sharing program). The RMFs were maintained as a monolayer in Dulbecco’s Modified Eagle’s Media (DMEM, Mediatech, Inc.) supplemented with 20% FBS. Laboratory strain B virus, E2490 (a gift from Dr. R. N. Hull) was used in the described experiments. All virus stocks were prepared and infectious virus quantified using Vero cells (Lot # CCL-81) obtained from ATCC®. Experiments with B virus were done in the BSL-4 maximum containment laboratory in accordance with the recommendations described in the Biosafety in Microbiological and Biomedical Laboratories manual (BMBL, 5^th^ edition).

### Antibodies and chemical compounds

Rabbit anti-human phospho-Akt (pAkt Ser-473) antibody, rabbit anti-human pan-Akt antibody, rabbit anti-human caspase-3 antibody, rabbit anti-human caspase-7 antibody, rabbit anti-human cleaved caspase-3 antibody, rabbit anti-human cleaved caspase-7 antibody, and rabbit anti-human GAPDH antibody were purchased from Cell Signaling Technology (Danvers, MA). Rabbit anti-human PARP antibody was purchased from Abcam (Cambridge, MA). Goat anti-rabbit IgG -HRP conjugated was purchased from Thermo Scientific (Waltham, MA). The PI3K inhibitor, LY294002, was purchased from Cell Signaling Technology (Danvers, MA). Sorbitol was purchased from US Biological Corporation (Boston, MA). Cyclohexamide (CHX), a eukaryotic protein biosynthesis inhibitor, purchased from Sigma-Aldrich Corp (St. Louis, Missouri).

### Western blot analysis

Both HFF or RMF cells were infected with B virus at a multiplicity of Infection (MOI) 5. Prepared infected cell lysates (with protease inhibitors) were collected in Laemmli sample buffer containing 5% 2- mercaptoethanol (v/v) (Bio-Rad, Hercules CA). The solution was boiled for 5 minutes and samples were fractionated using 10% SDS-PAGE, with gels subsequently immunoblotted to determine relative protein levels of pAkt (S473), pan-Akt, and GAPDH. A 15% SDS-PAGE gel was electrophoresed to fractionate samples and immunoblotted onto nitrocellulose (0.45 um) to determine relative protein levels of cleaved caspase-3 & -7, and cleaved-PARP. All antibodies from Cell Signaling were used at 1:1000 dilutions. The anti-PARP antibody was used at 1:400 and goat-anti rabbit IgG was used at 1:10,000 dilutions. All dilutions were made in Blotto™ containing 5% milk. Respective bands were detected using ECL Plus western blotting detection reagent (GE Healthcare Life Sciences, Piscataway NJ).

### Plaque assays

B virus infectious virus from RMF and HFF cells treated in the presence and absence of LY294002 was quantified using standard Vero cell plaque assays. Infected cells and supernatants in the presence and absence of LY294002 were collected 24 hours post infection (hpi). Infected cell lysates were frozen/thawed once, subsequently serially diluted (101–10^6^), and added to wells of a 24-well micro-well plate. Virus was adsorbed in a final volume of 100 μl for 1 hour at 37° C in a humidified 5% CO_2_ incubator. At 1 hour post adsorption medium was removed from each well, and fresh MEM medium containing 2% FBS mixed with 1% methylcellulose was added to the wells. The plates were incubated for 48 hpi, after which the medium overlay was removed, wells washed twice with PBS (Phosphate buffer saline), and fixed with 100% methanol. Fixed cells were stained with 0.2% crystal violet, and the number of plaques counted visually.

### Statistical analysis

In order to determine if there is significant difference between B virus titers in cells with PI3K inhibitor (LY294002) and cells without treatment, we performed Student’s t-test using B virus titers obtained from 3 independent experiments (n = 3). If the p-value was <0.05, then the two groups were found to be significantly different.

## Results

### B virus infection induces the phosphorylation of Akt at serine 473 in both macaque and human fibroblast cells

**Fig 1:**
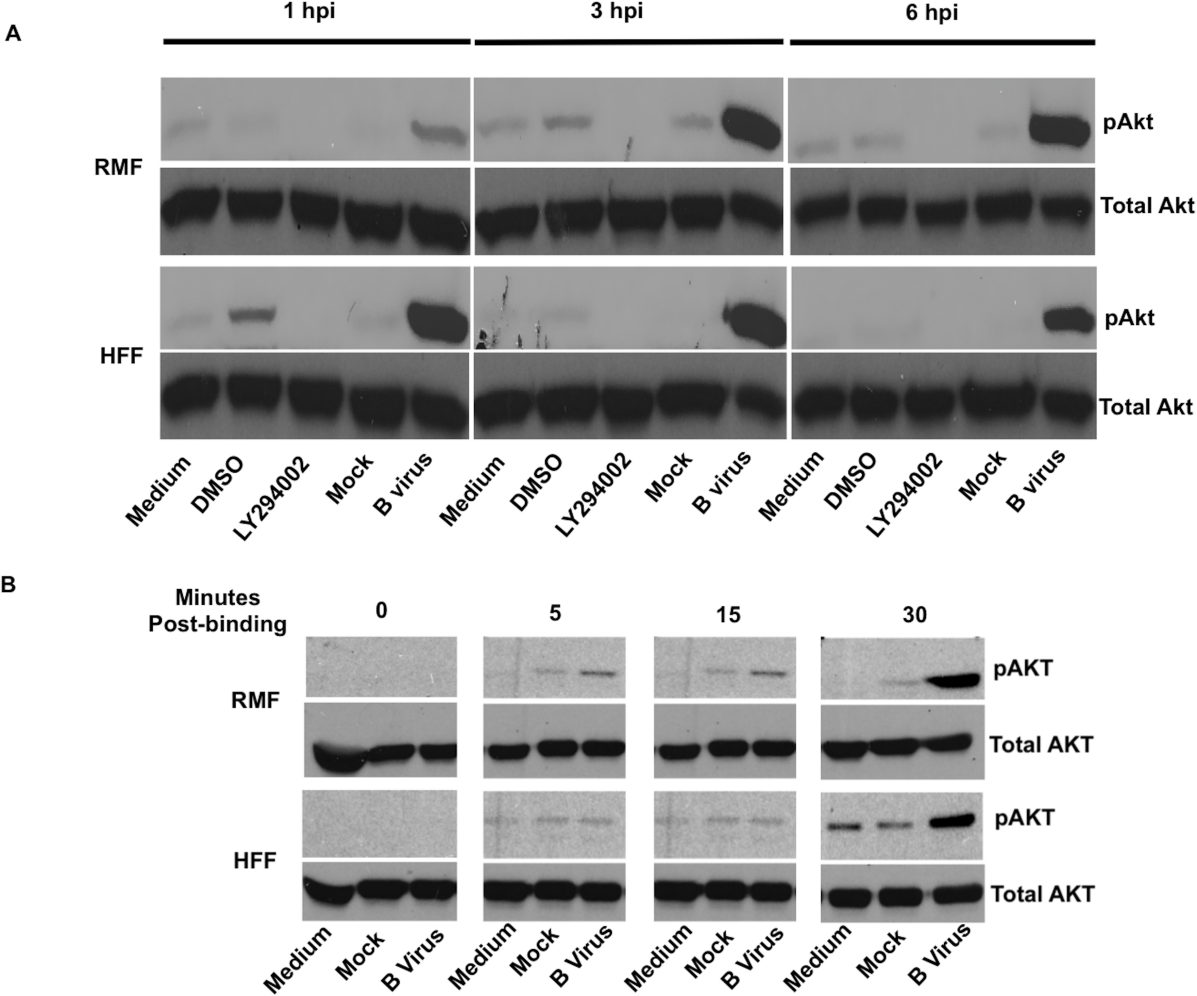
Phosphorylation of Akt in B virus infected macaque (RMF) and human cells (HFF). RMF and HFF cells were exposed either with B virus (MOI 5), mock-infected cell lysate (mock), following treatment with either 50 μM LY294002, DMSO, or medium for 1, 3, and 6 hpi. **(A)** Cell lysates were collected, fractionated, immunoblotted, and probed for pAkt (S473), using total Akt as a loading control. **(B)** Virus entry into RMF and HFF cells was synchronized by incubating at 4°C for 1 hour. After 1 hour adsorption at 4°C, cells were transferred to 37°C. Cell lysates were again prepared and probed for pAkt (S473) and total Akt at 0, 5, 15, and 30 minutes at 37°C.These results are representative of 3 independent experiments.

Subconfluent monolayers of each cell species (HFF and RMF) were infected by adsorption with B virus infected cell lysate (MOI 5) diluted in serum-free medium for one hour. Subsequently, medium was removed and infected cells were incubated in serum-free medium as described. For controls, cells were treated with plain medium, medium containing DMSO, LY294002 (50 μM), or medium containing mock-infected cell lysate. Cells were lysed at 1, 3, 6 hpi using Laemmli sample buffer containing 5% 2-mercaptoethanol. Cell lysates were fractionated and immunoblotted to assess relative levels of pAkt (S473), which is ultimately required for maximal kinase activity [29, 30]. Visual analysis of immunoblots showed that B virus infected cells phosphorylated Akt (S473) at all the measured time points in both macaque and human cells (Fig 1B). To determine whether Akt phosphorylation occurred immediately upon virus binding to the cell membrane, ice-cold virus was added to pre-chilled cells, and cells incubated for 1 hour at 4°C to maximize virus binding for ultimately synchronizing penetration. After 1-hour incubation at 4°C, infected cells were quickly transferred to 37°C to allow for virus penetration. Infected and control cell were harvested and lysed at 5, 15, 30 minutes post 37°C incubation to assess the earliest time at which pAkt (S473) was detectable. Infected cells were also collected and lysed at 0 hour at the time of transfer. Analysis of western blots revealed that B virus infected macaque cells show Akt (S473) phosphorylation within 5 minutes after being shifted to 37°C and infected human cells show this within 15 minutes after the temperature shift (Fig 1B). These results suggest that B virus infection of macaque cells and human cells correlates with virus binding to RMF and HFF cells, respectively, considering the slight delay observed in the appearance of pAkt in the HFF cells. While some basal levels of activation were apparent in mock infected cells, Akt (S473) was phosphorylated to a significantly greater extent in B virus infected macaque and human cells.

### B virus does not require *de novo* protein synthesis to initiate or continually phosphorylate Akt (S473) during the first 8 hpi in macaque and human fibroblast cells

**Fig 2:**
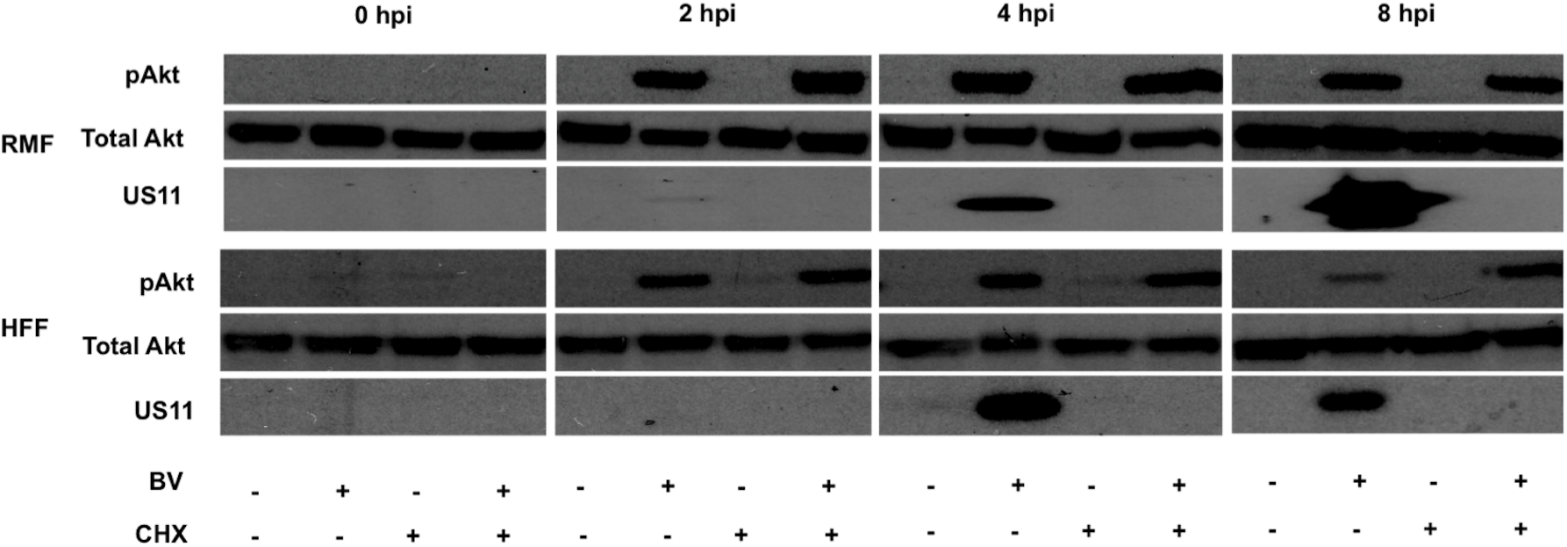
Phosphorylation of Akt in B virus infected macaque and human cells was independent of *de novo* protein synthesis. RMF and HFF cells were grown in the presence or absence of CHX (100 μg/ mL) in serum-free medium 1 hour prior to infection. Cells were infected with B virus (MOI 5) or mock cell lysate diluted in serum-free medium in the presence or absence of CHX. Cell were collected and lysed at 0, 2, 4, and 8 hpi, fractionated, immunoblotted, and probed for pAkt (S473), total Akt, and B virus Us11, the latter to validate CHX inhibition. These results are representative of 3 independent experiments.

Because B virus induced Akt phosphorylation was observed immediately following binding/entry, we next determined whether or not B virus infected cells required *de novo* protein synthesis to continually phosphorylate Akt (S473). To evaluate this, cells were grown in the presence or absence of cyclohexamide (CHX 100 μg/ mL) 1 hour prior to infection to inhibit translation. Cells were then infected (MOI 5) in serum-free medium in the presence or absence of CHX for 8 hours of infection. B virus infected macaque and human cells were observed to phosphorylate Akt (S473) regardless of whether or not protein translation occurred. To ensure effective inhibition of translation, we probed for Us11, an early protein expressed during B virus infection, which was absent in the CHX-treated cells (Fig 2). These data suggest that B virus structural proteins were sufficient to initiate and continue phosphorylation of Akt (S473) throughout infection in macaque as well as human cells targeted initially following infection.

### B virus phosphorylation of Akt (S473) is PI3K-dependent

**Fig 3:**
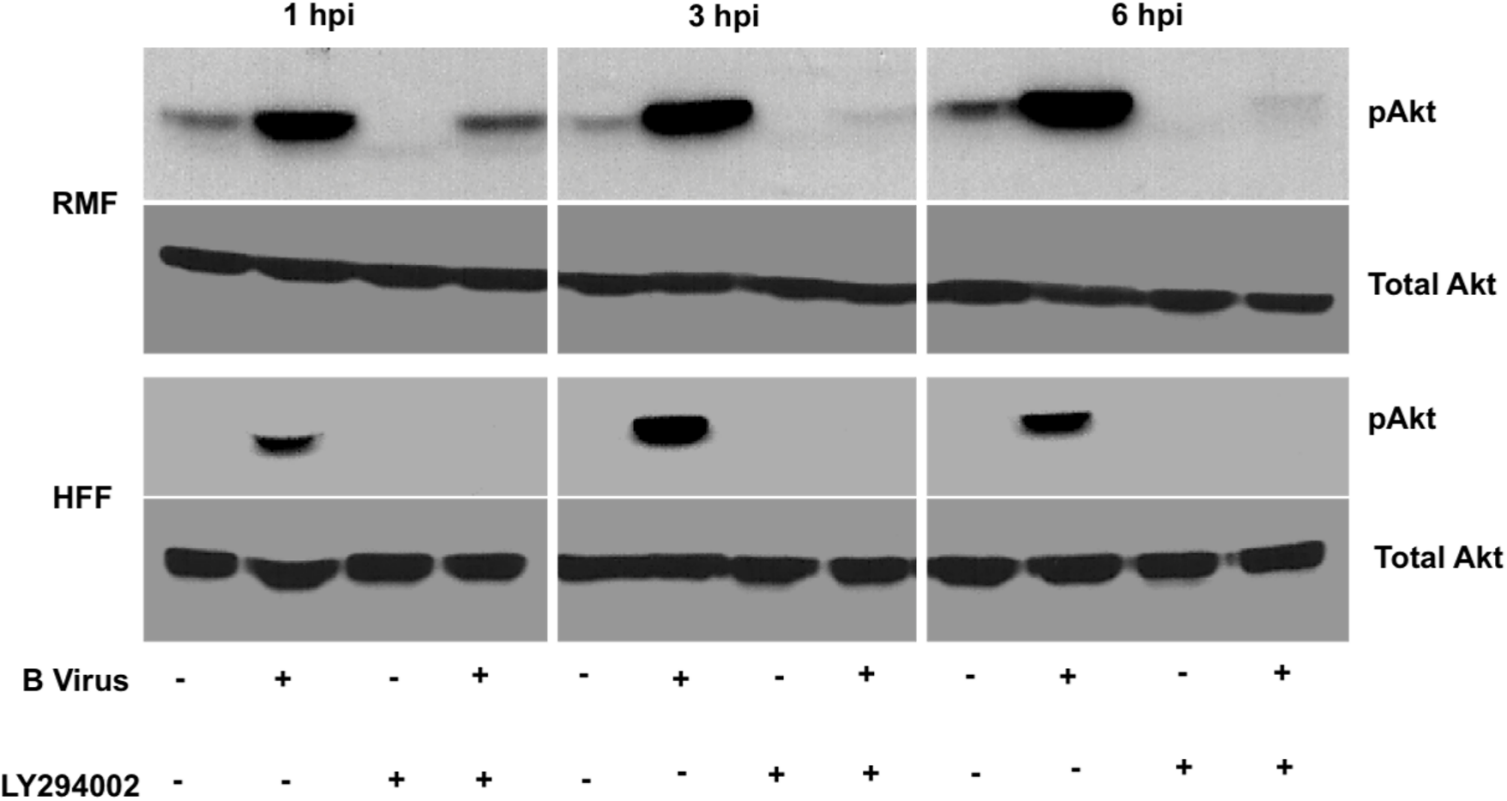
PI3K-dependent Akt phosphorylation in B virus infected cells. RMF and HFF cells were either grown in the presence or absence of 50 μM LY294002 for 2 hours prior to infection and throughout the duration of infection. Cells were collected at 1, 3, 6 hpi, lysed, fractionated, immunoblotted, then probed for pAkt (S473) and total Akt. These results are representative of 3 independent experiments.

Akt activation occurs in a PI3K-dependent or–independent manner. In order to validate whether B virus infection-induced phosphorylation of Akt (S473) was PI3K-dependent, we treated RMF and HFF cells with 50 μM LY294002 for 2 hours prior to infection (MOI 5) and throughout 6 hpi. Results from this experiment showed that upon treatment with LY294002, B virus failed to induced Akt (S473) phosphorylation was detected in HFF cells at any time suggesting that B virus-induced Akt (S473) phosphorylation was PI3K-dependent (Fig 3). In RMF cells, LY294002 treatment prevented B virus-induced Akt (S473) phosphorylation completely by 3 hpi, however, at 1 hpi Akt (S473) phosphorylation was detected even in the presence of LY294002 (Fig 3). These results suggest that B virus phosphorylation of Akt (S473) initially appears to be PI3K-independent in RMF cells, but later becomes predominantly PI3K-dependent, as previously noted in studies that demonstrated phosphorylation of S473 increases rapidly via a PI3K-dependent process following stimulation.

### PI3K-dependent Akt (S473) phosphorylation stimulates virus replication in RMF cells, but not in HFF cells

**Fig 4:**
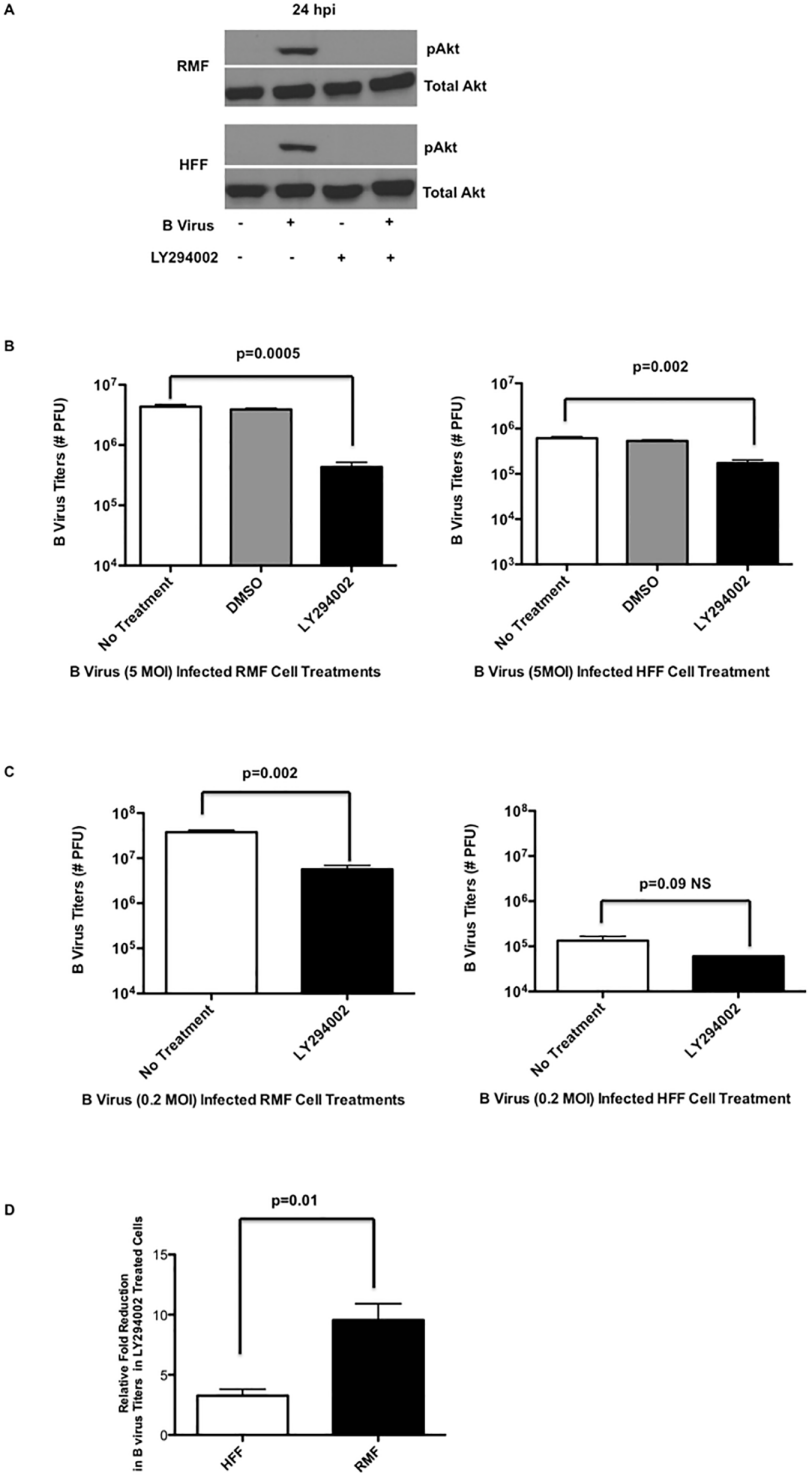
Effect of PI3K/Akt activation on B virus replication in human and macaque cells. RMF and HFF cells were grown in the presence or absence of LY294002 for 2 hours prior to infection and the treatment was continued throughout the infection for 24 hours. **(A)** Cell lysates were collected at 24 hpi and the effect of LY294002 on phosphorylation of Akt (S473) was assessed. **(B)** B virus titers in infected (MOI 5) RMF/HFF cells with or without LY294002 treatment. **(C)** B virus infectious titers in infected (MOI 0.2) RMF/HFF cells with or without LY294002 treatment. **(D)** Relative fold-reduction in infectious virus titers in RMF and HFF cells treated with LY294002. All results are representative of 3 independent experiments.

We examined whether or not inhibition of PI3K-dependent phosphorylation of Akt (S473) affected B virus replication in either RMF or HFF cells. We grew RMF and HFF cells in the serum-free medium, medium containing DMSO, or medium containing 50 μM LY294002 for 2 hours prior to infection, after which cells were infected with B virus at a MOI 5. Cells treated with LY294002 throughout the infection for 24 hours. Analysis of western blot results demonstrated that B virus infection resulted in phosphorylation of Akt (S473) throughout 24 hpi, however, Akt phosphorylation of S473 was absent when PI3K was inhibited in each cell line (Fig 4A). To examine whether PI3K was critical for efficient virus replication, infected cells and supernatants were collected, frozen/thawed and plaque-assayed to quantify infectious B virus titers. The results of this experiment showed that in the absence of Akt (S473) phosphorylation in RMF cells, virus titers were significantly reduced (1 log reduction; p = 0.0005) relative to controls; Although B virus titers in HFF cells treated with LY294002 were reduced significantly relative to controls (p = 0.002), this reduction was only 3 times (0.3 log) less when compared to controls (Fig 4B). To further verify the magnitude of this reduction in RMF vs HFF cells, we infected the cells treated with or without LY294002 with lower MOI (0.2). Our results indicated that the virus titers in RMF cells were significantly reduced (p = 0.002; 0.7 log reduction) in the presence of PI3K inhibitor when compared to virus titers in untreated RMF cells. Interestingly, at a lower MOI, B virus titers in HFF cells during PI3K inhibition showed no significant difference (p = 0.09, ~0.2 log reduction) (Fig 4C) when compared to B virus infecte cells without PI3K inhibition. Moreover, the relative-fold reduction in B virus titers with PI3K inhibition was significantly different between RMF and HFF cells (Fig 4D). Our results, thus, demonstrated that although Akt (S473) was phosphorylated in each cell line, efficient B virus replication in RMF cells was dependent on PI3K phosphorylation of Akt (S473). Although B virus replication efficiency in HFF cells was affected when PI3K dependent Akt (S473) phosphorylation was blocked, it was affected to a lesser extent compared to RMF cells. The relative fold-reduction in virus titers between cell lines, HFF and RMF, indicate a significant difference, when PI3K was inhibited, however only a modest difference between the cell lines was noted (Fig 4D).

### B virus infected RMF cells require PI3K -induced Akt (S473) phosphorylation to block the expression of apoptotic markers

**Fig 5:**
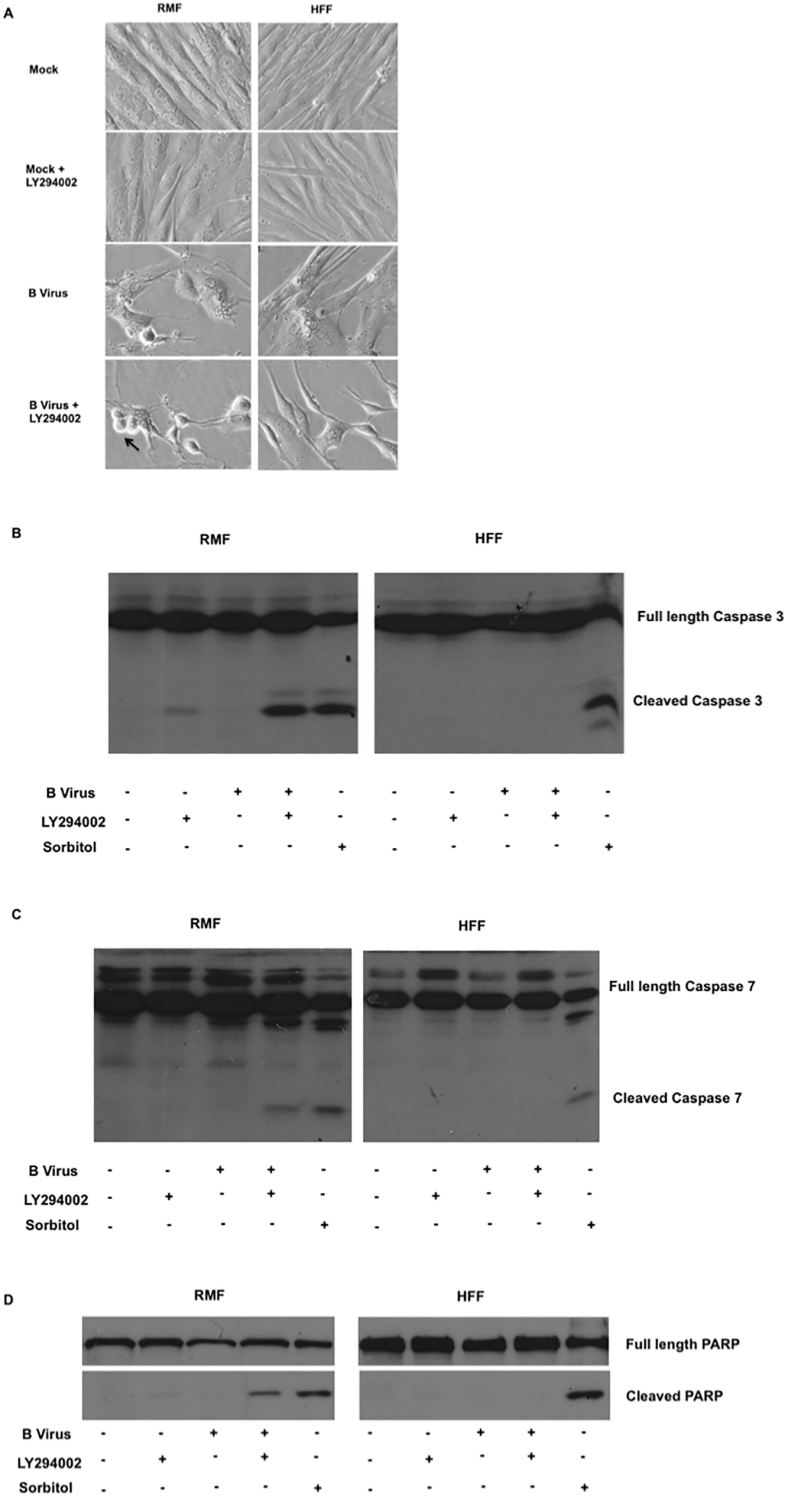
Expression of apoptotic markers in B virus infected macaque and human cells in the absence of PI3K/Akt activation. RMF and HFF cells were treated in the presence and absence of LY294002 2 hours prior to infection and throughout infection for 24 hours. **(A)** Cell morphology was observed microscopically. **(B)** At 24 hpi cells were collected, fractionated, immunoblotted, and probed for cleaved caspase-3, **(C)** cleaved caspase-7, and **(D)** cleaved PARP. These results are representative of 3 independent experiments.

To confirm that one function of PI3K is to block apoptosis, experiments were performed in which this PI3K was prevented from activating Akt (S473). In the absence of functional PI3K, B virus titers were exponentially reduced in RMF cells. We hypothesized that in B virus infected RMF cells, apoptosis was blocked via PI3K activation of Akt (S473). To test this, we infected RMF and HFF cells in the presence and absence LY294002 treatment. Mock-infected cells grown in the presence and absence of LY294002 provided a negative control, while the positive control consisted of RMF and HFF cells treated with 1.5 M sorbitol for 4 hours to induce apoptosis. Sorbitol treatment of cells causes osmotic stress resulting in apoptosis. This effect of sorbitol is independent of PI3K/Akt, but treated cells express similar apoptotic markers. This functionality of sorbitol makes it a good positive control for apoptosis. At 24 hpi or 4 hours post sorbitol treatment, cells were observed microscopically to assess cell morphology. We noted that only B virus infected RMF cells treated with LY294002 revealed microscopic apoptotic characteristics, including morphological changes in the nucleus, cell rounding, and surface blebbing (Fig 5A). To verify whether the morphological changes were indeed due to apoptosis, we quantified apoptotic markers, including cleaved forms of executioner caspases -3 (Fig 5B, Sup Fig 1A) and -7 (Fig 5C, Sup Fig 1B) along with the cleaved form of PARP (Fig 5D, Sup Fig 1C). Our results show that only B virus infected RMF cells treated with LY294002 express intrinsic apoptotic markers (Fig 5B–5D). Thus, these data suggest that B virus infected macaque RMF cells express intrinsic apoptotic markers when Akt (S473) activation via PI3K is blocked, unlike HFF cells that failed to express apoptotic markers under the experimental conditions explored.

## Discussion

Individuals working directly with macaques or with macaque samples are at risk of contracting B virus with deadly consequences in the absence of effective intervention. It is very interesting to note that a seemingly harmless virus in its natural host can wreak havoc in a foreign host such as humans particularly given the innocuous infection in humans caused by the closely related HSV types 1 and 2. Rationally, therefore, understanding the differences that exist in the virus: host cell interactions between different species help in developing interventions to control the deadly B virus infection. Herpes virus infection at the site of entry results in modulation of host-specific defense signaling pathways that are vulnerable to being hijacked to promote efficient virus replication. To this end herpes viruses are known to activate several signaling pathways involved in cell survival such as MAPK/ERK, PI3K/Akt pathways, and inhibit apoptotic and antiviral pathways. Activating the cellular PI3K/Akt pathway drives the infected cell toward cell survival thwarting protection of the host that results from cell death via apoptosis, thus, forcing the cell to remain viable to support virus replication and spread. RNA viruses, such as influenza virus, vesicular stomatitis virus, poliovirus, Ebola virus etc., also induce Akt activation [31–33]. Almost all herpes viruses are known to activate Akt in infected cells. Independent research groups have shown that HSV activates Akt to facilitate entry, inhibit apoptosis, and aid in efficient virus replication. Therefore, the Akt signaling pathway is considered to be a major pathway to target to enable therapeutic strategies to suppress HSV infection. Beta herpes viruses, such as human cytomegalovirus (HCMV), murine cytomegalovirus (MCMV) etc., activate Akt to block apoptosis of the infected cells. Kaposi’s sarcoma associated herpesvirus (KSHV), a gamma herpesvirus, also activates Akt for lytic cycle replication. Epstein-Barr virus (EBV) another gamma herpesvirus utilizes Akt induction for virus growth and survival. Here we explored PI3K-dependent Akt activation during B virus infection of macaque (RMF) and human (HFF) cells to evaluate whether Akt activation plays an important role in efficiency of virus replication and blocking apoptosis and to study whether natural and foreign host cell differed in terms of the role(s) of Akt activation. Our results clearly suggest that B virus activates Akt as early as 5–15 minutes after virus binding to cells (Fig 1B) and this activation continues throughout B virus infection (Figs 1A and 2) of dermal fibroblasts regardless of whether macaque or human cells are used. Early activation of Akt after B virus infection suggests that the initial activation of Akt during B virus is probably mediated by B virus glycoprotein binding to receptors on the cell surface. The extended activation of Akt in the absence of viral *de novo* protein synthesis (Fig 2) also suggests that B virus likely engages more than one viral protein to fully induce Akt activation in infected cells.

Akt is activated predominantly via PI3K, but there are other mechanisms that activate Akt independently of PI3K. For instance I-kappa B-kinase epsilon (IKBKE) [34], TANK-binding kinase-1 (TBK1) [35], and DNA-PKcs proteins [36] directly activate Akt independently of PI3K. Here we show for the first time that B virus infected cells activate Akt in a PI3K-dependent manner, however, in cells derived from the natural hosts, macaques, Akt can also be activated independently of PI3K at least for the first 3 hpi, though less efficiently than during PI3K-dependent activation, as shown in the blocking experiments presented. There were no significant differences between RMF and HFF Akt activation pathways, and indeed, we observed Akt activation (S473) in both cell lines. To better understand the impact of this activation, we blocked the process to see the impact of this pathway on virus replication. Significant reduction in virus replication titers was observed in both RMF and HFF cells when Akt activation was blocked; however, this reduction was slightly greater in RMF cells compared to HFF cells. We therefore hypothesize that Akt plays an important role in inhibiting apoptosis and promoting virus replication in RMF cells.

It is important to note that cells that are not stressed do not initiate apoptosis when Akt activation is inhibited. Activation of Akt can inhibit pro-apoptotic molecules (Bad, FOXO-1) [37, 38] and activate anti-apoptotic protein molecules, such as X-linked inhibitor of apoptosis (XIAP) that are involved in the intrinsic apoptotic pathway [39]. Subsequently, XIAP in turn inhibits both caspase -3 and -7 although the mode of inhibition is different for each caspase [40, 41]. Cleaved caspase-3 and -7 also function in cleaving the full length PARP (~116 KDa) to ~89 KDa and ~24 KDa fragments [42] in order to prevent PARP from trying to repair the nicks in DNA due to DNA fragmentation during apoptosis. To this end, our results demonstrated that preventing Akt activation during B virus infection of macaque fibroblast cells results in activation of intrinsic apoptosis as evidenced by the cleavage of caspase-3,-7, and PARP. In case of B virus infected HFF cells, PI3K/Akt activation may or may not block apoptosis, since in the absence of PI3K/Akt activation, the infected cells failed to undergo apoptosis. These results suggest that in human HFF cells B virus might engage in an alternative signaling pathway prevents apoptosis in the absence of PI3K/Akt induction. The RMF cells are macaque-derived adult dermal fibroblast cells whereas the human fibroblast cells were derived from neonatal foreskin. Although adult cells are more prone to Fas-FasL dependent apoptosis no difference was apparent in Fas-FasL indepenedent apoptosis [43]. Because in our studies there is no interaction of Fas-FasL, the apoptotic markers we observed in B virus infected macaque cells treated with LY294002 were due to the stress caused by the virus in the absence of PI3K/Akt activation. This conclusion is further corroborated by the absence of apoptotic markers in mock-infected cells treated with or without LY294002 treatment (S1 Fig). Human HSV types -1 & -2 infect human HFF cells very effectively, paralleling herpes B virus infection in macaque cells. Interestingly, however, macaque cells are not susceptible to human HSV types -1 &-2 [44–46]. From studies on HSV-1 and HSV-2 infection in rhesus macaques and macaque cells along with our data presented here, we hypothesize that there are likely significant differences in signaling pathways in B virus infected natural hosts when compared to foreign hosts. The differences we observed in the two different cell lines, however, are not necessarily a representation of *in vivo* infections in the respective species and as such further studies are required to validate whether these changes are indeed a reflection of what occurs in macaque and human species during B virus infection.

## Supporting information

S1 Fig Densitometric analysis of expression of apoptotic markers in macaque and human cells. Cleaved caspase -3, cleaved caspase -7 and cleaved PARP bands were normalized using total caspase -3, caspase -7, and PARP. **(S1A)** Fold-difference in the expression of cleaved caspase-3 relative to mock infected RMF and HFF cells. **(S1B)** Fold-difference in the expression of cleaved caspase-7 relative to mock infected RMF and HFF cells. **(S1C)** Fold-difference in the expression of cleaved PARP relative to mock infected RMF and HFF cells.
(TIFF)
