# Supplementary figures and images for: Regulation of PI3K/Akt dependent apoptotic markers during b virus infection of human and macaque fibroblasts
Source: PLoS One. 2017 May 30;12(5):e0178314. doi: 10.1371/journal.pone.0178314 (PMC5448769; doi:10.1371/journal.pone.0178314)

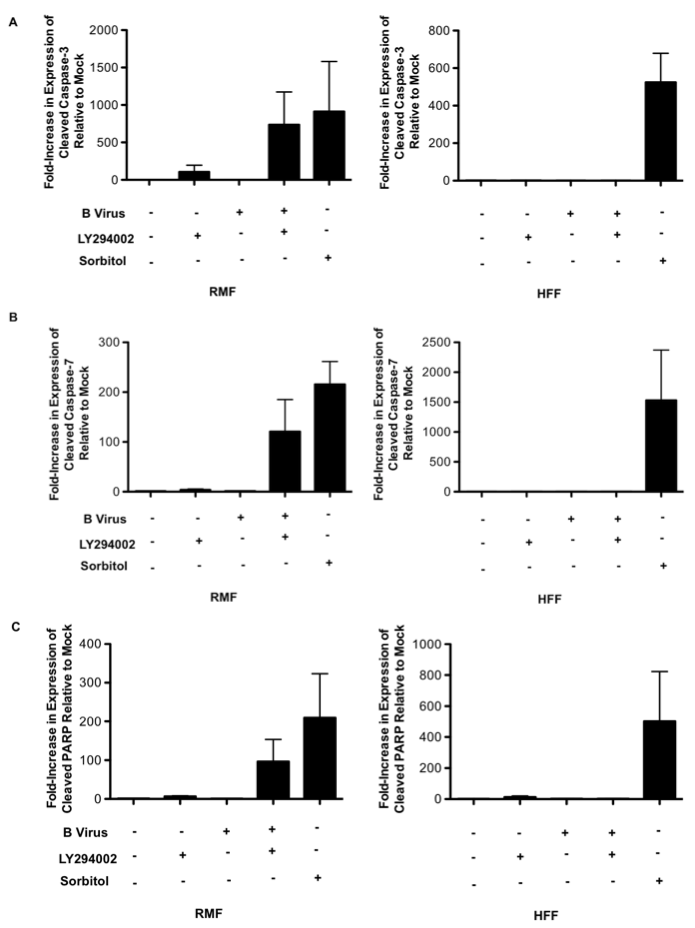

Supplement: S1 Fig — Cleaved caspase -3, cleaved caspase -7 and cleaved PARP bands were normalized using total caspase -3, caspase -7, and PARP. (S1A) Fold-difference in the expression of cleaved caspase-3 relative to mock infected RMF and HFF cells. (S1B) Fold-difference in the expression of cleaved caspase-7 relative to mock infected RMF and HFF cells. (S1C) Fold-difference in the expression of cleaved PARP relative to mock infected RMF and HFF cells. (TIFF) [file pone.0178314.s001.tiff]
